# Editorial: Glucosinolate Metabolites: Bioavailability, Bioactivity and Clinical Variability

**DOI:** 10.3389/fnut.2021.823203

**Published:** 2021-12-21

**Authors:** Donato Angelino, Elizabeth H. Jeffery

**Affiliations:** ^1^Faculty of Bioscience and Technology for Food, Agriculture and Environment, University of Teramo, Teramo, Italy; ^2^Department of Food Science and Human Nutrition, University of Illinois, Urbana, IL, United States

**Keywords:** glucosinolate, isothiocyanate, bioavailability, bioactivity, gut microbiota

Health benefits from inclusion of brassica or brassica components in the diet have intrigued scientists for well over 25 years, yet we still flounder in determining how to best provide those benefits to the general public, or even to prove the point to the scientific community. At first considered as chemopreventive agents, acting by enhancing excretion of foreign compounds, it has become apparent that brassica bioactives can impact our health in multiple ways. Maybe the most impactful manner is by inhibiting inflammation, known to aggravate many health concerns including most non-contagious diseases. The list of health benefits has thus grown, now including a positive impact on diseases such as diabetes, heart disease and, most recently, several cognitive diseases (as pointed out by Marino et al.). Unfortunately, many clinical trials have failed to confirm health benefits seen in animal studies. A major problem appears to be variation in response among subjects. The cause for this is addressed in every manuscript in this series. In contrast, cell culture studies show mechanism clearly. Typically, preclinical studies in rodents show efficacy and even epidemiological studies often suggest efficacy (see the manuscript by Wei et al.), but clinical studies frequently show so much variability among subjects that nothing can be confirmed. Maybe in part for this reason, many clinical studies are still focused on bioavailability after almost three decades: see Charron et al. in this issue, who identified a role for BMI in variability of plasma GSL metabolite abundance, among subjects in a clinical trial. All the papers in this series address this problem of reproducibility in clinical studies–and today's overriding hypothesis for this variability appears to be individual variation in the gut microbiome.

To address this problem, two papers review aspects relating to the set-up and development of published human clinical trials involving glucosinolates (GSL), their bioactive isothiocyanates (ITC) metabolites and GSL-containing vegetables, with discussions that look toward future design of clinical trials. Fahey and Kensler explain “blow by blow” all the difficulties they overcame in development of a “broccoli sprouts soup” for use in clinical trials. The sprouts were utilized both for their greater concentration of bioactives compared to the mature vegetable and for their use in formulation of a consistent product, that they have used for a number of clinical studies. These authors discuss that, as the GSL metabolite sulforaphane (SFN) is considered responsible by many for the beneficial activities of broccoli, maybe the “simple” administration of SFN might outflank the multiple drawbacks in controlling broccoli cooking and digestive processes. However, they conclude that, unlike other bioactives, supplementation with SFN is unfortunately not so simple due to its chemical instability. Furthermore, it would be less advantageous nutritionally, leading to a decrease in broccoli consumption and other bioactives and nutrients associated with dietary broccoli. For these reasons, clinical trials often use whole broccoli or glucoraphanin with or without myrosinase, making uncertain the exact dose of GSL-derived metabolites delivered. As they point out, there is still much to understand if we are to overcome the wide variation among individual subjects' responses, necessary to both prove that health benefits extend to humans and ensure that broccoli and broccoli products provide benefits to the public.

The paper by Marino et al. lists clinical trials from the NIH database “ClinicalTrials.gov” from 2000 to 2020 that include GSLs, GSL metabolites or GSL-containing vegetables. Major characteristics and findings are tabulated, providing a useful database for those of us in the field. Most of the 87 published studies retrieved focus on GSL-rich extracts or components, rather than on whole foods, mostly from broccoli. Primary outcomes covered multiple health effects, with most studies focused on either cognitive function or cancer. Intriguingly, only two clinical studies not yet published (1, 2) and one published clinical study (3) considered the interactive impact of GSL with the gut microbiome, although such an interaction is a key point of discussion for the majority of manuscripts throughout this special issue.

In contrast, the review by Ho and colleagues provides a major focus on the gut microbial species able to convert GSL into ITC, indoles, and other metabolites. The authors highlight those few studies of metabolism of GSL by the human microbiome: to date, these are *ex vivo* using fecal cultures. However, there is a growing number of animal-derived microbiome studies of GSL metabolism, both *in vivo* and *ex vivo*. Among the species found to convert GLS into bioactive ITC (and/or nitriles), most identified belong to the *Enterococcus, Bifidobacterium, Lactobacillus* and *Lactococcus* genera. The authors also highlight the importance of the diet and its impact on the presence/persistence of such species.

Two studies consider the role of GSL and their metabolites in cancer chemoprevention and survival. In one, Williams discusses glucobrassicin metabolism and efficacy, focusing on the formation of the key metabolites indole-3-carbinol and 3,3'-diindolylmethane and their inhibitory impact on a variety of pathways in cancer development, mostly citing cell culture studies. Pathways impacted include detoxification, tumor growth, estrogen metabolism and others. Interestingly, Williams concludes that biologically significant doses of these metabolites can be reached through the consumption of Brussels sprouts and other brassica in a normal diet. Furthermore, like others in this series, he proposes that the gut microbiome may play an important role in variability among individual responses.

In an epidemiological approach, Wei et al. evaluate the role of brassica vegetables and ITC intakes in survival following ovarian cancer, in a hospital-based cohort of Chinese subjects. Encouragingly, subjects within the highest tertile of brassica intake (>70 g/day, plasma ITC ≥9.44 μmol/day), exhibited improved survival compared to those in the lowest tertile of intake (<33 g/day brassica intake, ≥3.90 μmol ITC/day).

The paper by Charron et al., a clinical bioavailability study, found that the Body Mass Index (BMI) of an individual may offer a key to variability of GSL metabolites seen after consumption of cooked broccoli. Plasma levels from subjects with a BMI >26 kg/m^2^ show earlier and greater (double) appearance of GSL metabolites in individuals with a BMI below 25 kg/m^2^. In considering known differences in gut microbiota species with BMI (*Firmicutes*/*Bacteroides* ratio is elevated in obesity), they suggest that this may impact GSL metabolite bioavailability, leading to a possible difference in efficacy (no efficacy endpoint was evaluated).

Is this interest in the interaction between GSL and the microbiome just the latest hot topic, or this time have we finally arrived at the source of variability of clinical response to GSL? If so, what should our strategies be for overcoming this or successfully handling the data—and will we finally be able to successfully offer brassica to the general public in a manner that will improve health for most people? As new tools allow a more complete evaluation of an individual's microbiome, it may become possible to propose personalized nutrition to either alter the microbiome make-up with prebiotics, or to advise the frequency of broccoli intake necessary for the individual to gain health benefits.

## Author Contributions

EJ wrote the introduction and the conclusion. DA wrote the central part with comments to the cited papers and references. Both authors contributed to the article and approved the submitted version.

## Conflict of Interest

The authors declare that the research was conducted in the absence of any commercial or financial relationships that could be construed as a potential conflict of interest. The handling Editor (MS) declared a shared affiliation with one of the authors (DA) at the time of review.

## Publisher's Note

All claims expressed in this article are solely those of the authors and do not necessarily represent those of their affiliated organizations, or those of the publisher, the editors and the reviewers. Any product that may be evaluated in this article, or claim that may be made by its manufacturer, is not guaranteed or endorsed by the publisher.

## References

[1] Effects of Brassica on Human Gut Lactobacilli (EBL). (2015). Available online at: https://clinicaltrials.gov/ct2/show/NCT02291328 (accessed November 25, 2021).

[2] Discovery of Biological Signatures for Cruciferous Vegetable Intake (Single Serving). (2021). Available online at: https://clinicaltrials.gov/show/NCT04641026 (accessed November 25, 2021).

[3] Kellingray L Tapp HS Saha S Doleman JF Narbad A Mithen RF. Consumption of a diet rich in Brassica vegetables is associated with a reduced abundance of sulphate-reducing bacteria: a randomised crossover study. Mol Nutr Food Res. (2017) 61:1600992. 10.1002/mnfr.201600992 28296348 PMC5600105

